# Manipulating disordered plasmonic systems by external cavity with transition from broadband absorption to reconfigurable reflection

**DOI:** 10.1038/s41467-020-15349-y

**Published:** 2020-03-24

**Authors:** Peng Mao, Changxu Liu, Fengqi Song, Min Han, Stefan A. Maier, Shuang Zhang

**Affiliations:** 1 School of Physics and Astronomy, University of Birmingham, Birmingham, B15 2TT UK; 2 College of Electronic and Optical Engineering & College of Microelectronics, Nanjing University of Posts and Telecommunications, 210023 Nanjing, China; 3 Chair in Hybrid Nanosystems, Nanoinstitute Munich, Faculty of Physics, Ludwig Maximilians University of Munich, 80539 Munich, Germany; 4 National Laboratory of Solid State Microstructures, Collaborative Innovation Center of Advanced Microstructures, and College of Engineering and Applied Sciences, Nanjing University, 210093 Nanjing, China; 5 Department of Physics, Imperial College London, London, SW7 2AZ UK

**Keywords:** Optical materials and structures, Nanophotonics and plasmonics

## Abstract

Disordered biostructures are ubiquitous in nature, usually generating white or black colours due to their broadband optical response and robustness to perturbations. Through judicious design, disordered nanostructures have been realised in artificial systems, with unique properties for light localisation, photon transportation and energy harvesting. On the other hand, the tunability of disordered systems with a broadband response has been scarcely explored. Here, we achieve the controlled manipulation of disordered plasmonic systems, realising the transition from broadband absorption to tunable reflection through deterministic control of the coupling to an external cavity. Starting from a generalised model, we realise disordered systems composed of plasmonic nanoclusters that either operate as a broadband absorber or with a reconfigurable reflection band throughout the visible. Not limited to its significance for the further understanding of the physics of disorder, our disordered plasmonic system provides a novel platform for various practical application such as structural colour patterning.

## Abstract (web summary)

The tunability of disordered systems with a broadband response has not been explored. Here, the authors achieve the controlled manipulation of disordered plasmonic systems, realising a transition from broadband absorption to tunable reflection through control of the coupling to an external cavity.

## Introduction

With recent advances in nanophotonics and nanofabrication, disordered nanostructures are employed in different optical systems with unique features that otherwise cannot be realised by their periodic counterpart, including unconventional intensity statistics^1–3^, broadband transmission enhancement^4,5^, perfect focusing^6,7^, broadband light trapping^8^ and broadband energy harvesting^9–11^. By virtue of the intrinsic nature of the disorder, the macroscopic optical properties of such systems are robust to perturbations and variations of initial conditions^11,12^. As an example, nanoparticles dispersed in a solution have been considered for either energy harvesting or random lasing^13^. Despite the Brownian motion of the nanoparticles, which dynamically varies the spatial electric field profile, the statistical properties such as the transmission, intensity distribution and lasing wavelength are independent of fluctuations and insensitive to polarisation or variation in incident angle of the light source. The robustness to perturbations and initial conditions naturally provides disordered systems with a broadband optical response, which serves as one of the most prominent and beneficial factors, not only in artificial photonic systems^4,5,8–12,14,15^ but also in biosystems such as the white scale of *Cyphochilus* beetles^16^, white skin on Pyjama squids^17^ or black feathers from birds of paradise^18^. On the other hand, few efforts have been devoted to explore the tunability of disordered systems with a broadband response.

In this paper, we demonstrate the reconfigurability of a disordered plasmonic system through an external cavity underneath as an optical background for manipulating the decay rate of a specific mode. Counterintuitively, through deterministic control of the thickness of the external cavity without absorption, the disordered plasmonic system experiences a phase transition, from a system with broadband absorption to one with finite reflection bands. In the latter scenario, the reflection peaks can be continuously tuned through the whole visible spectrum. Besides theoretical analysis based on coupled mode theory (CMT) and ab initio full-wave simulations, we experimentally realise a hybrid system with either broadband absorption (>90% in the visible) or reconfigurable reflection bands with peaks ranging from 400 to 750 nm on the same platform. Our disordered plasmonic system provides a novel mechanism for a plethora of applications. As a proof-of-concept example, we demonstrate a broad range of structural colours without employing sophisticated and/or time-consuming lithography processes.

## Results

### Sample design and simulations

**Fig. 1:**
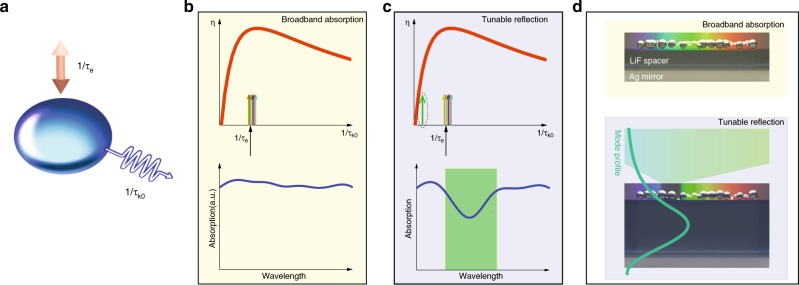
A disordered system with transition from broadband absorption to band-limited reflection/transmission based on coupled mode theory. **a** Schematic for a generalised optical cavity with coupling rate *τ*_*e*_ and intrinsic decay rate *τ*_*k*0_ for the *k*^th^ mode supported. **b** Broadband absorption regime. Upper panel: the disorder induces the convergence of intrinsic decay rates, leading to energy equipartition with similar coupling efficiency *η* for all the modes. Lower panel: broad absorption is achieved, computed with 25 modes with *τ*_*k*0_ converging within [0.8, 1.2]*τ*_*e*_. **c** Band-limited regime. Upper panel: sudden reduction of intrinsic loss of a specific mode; lower panel: an absorption dip is formed by enlarging the intrinsic decay *τ*_*k*0_ (by 20) of specific mode. **d** A realistic design matching the CMT model, composed of a disordered plasmonic system on a cavity with plasmonic mirror beneath. The thickness *t* can tune the intrinsic decay rate of a specific mode, driving the system from a broadband absorption (upper panel, *t* is too small to hold a mode) to band-limited reflection (upper panel, with a mode confined around the plasmonic structures).

We first consider a generalised model composed of a simple optical cavity, with coupling rate 1∕*τ*_*e*_ and intrinsic decay rate 1∕*τ*_*k*0_ for the *k*^th^ mode supported by the system, as illustrated in Fig. 1a. Based on CMT, the coupling efficiency for the *k*^th^ mode to the cavity can be expressed as the following:

1 $$\eta =\frac{2{\tau }_{e}/{\tau }_{k0}}{{(1+{\tau }_{e}/{\tau }_{k0})}^{2}}$$

with details shown in Supplementary Note 1 and ref. ^10^. The optimised coupling (absorption) occurs when the two rates match each other, similar to impedance matching in the electronic systems. For an optical system with intense disorder, the decay rates (1∕*τ*_*k*0_) converge to a constant and consequently the optical energy can be equally distributed to each mode^10^. Such energy equipartition mechanism equips the disordered system with a broadband response, as shown in Fig. 1b. The upper panel illustrates the situation when the decay rates (colourful arrows) matches the coupling rate (black arrow), leading to high coupling efficiency for all the modes. Consequently, a broadband absorption is achieved, as shown in the lower panel in Fig. 1b. Interestingly, the system can transit to another regime with band-limited response by a simple operation. As illustrated in Fig. 1c, when the decay rate of a specific mode is suddenly reduced, a mismatch between 1∕*τ*_*e*_ (black arrow) and 1∕*τ*_*k*0_ (green arrow) is induced with a significant reduction of the coupling efficiency for that mode (upper panel). As a result, the disordered system cannot hold (absorb) that mode but must release it, forming a band-limited reflection or transmision (as shown in the lower panel). In addition, the freedom of selecting modes in different spectral position allows tunability of the system, producing absorption dip at desired position.

Inspired by this model, we design a reconfigurable disordered system based on a disordered assembly of plasmonic nanoparticles on top of a planar optical cavity, as demonstrated in Fig. 1d. We choose the material of the cavity as a transparent (lossless) one, so that the light trapped inside would not be dissipated. The plasmonic system is composed of nanometre-sized silver particles with randomised size and position, providing the required disorder. The nanoparticles are deposited on a dielectric spacer above a silver substrate, forming a Fabry-Perot-liked system. A plasmonic mirror is introduced at the backside to improve the confinement of the cavity. More importantly, such a mirror plays an indispensable role to enhance the light-matter interactions. Together with the metallic nanoparticles on top, it reflects the photons and lets them be absorbed by the plasmonic network (more details in Supplementary Note 4). When the spacer thickness is too small to support any Fabry-Perot mode in the visible, the randomised nanoparticles perform as a system with broadband absorption as shown in the upper panel of Fig. 1d, owing to the disorder-induced energy equipartition. The system transforms to another regime if a certain optical mode can be confined, as depicted in the lower panel of Fig. 1d. The Fabry-Perot structure behaves like an external optical environment that can tune the optical response of the system^19,20^. Here, the configuration of a Fabry-Perot cavity constrains a specific mode inside the dielectric with tiny loss, reducing the absorption of the nanostructures, or equivalently reducing the intrinsic decay rate 1∕*τ*_*k*0_ for the target mode. Owing to the mismatch with the coupling rate, this mode will be reflected instead of being absorbed, generating a band-limited response as desired. Controllability is readily achieved by straightforwardly tuning the spacer thickness to release the light in particular modes. From another point of view, the disordered system reverses the reflection property of the cavity. The photons preserved in the cavity is reflected back when other photons are absorbed by the disordered system, while a pure Fabry-Perot cavity always captures (absorbs) the photons around its resonance.

**Fig. 2:**
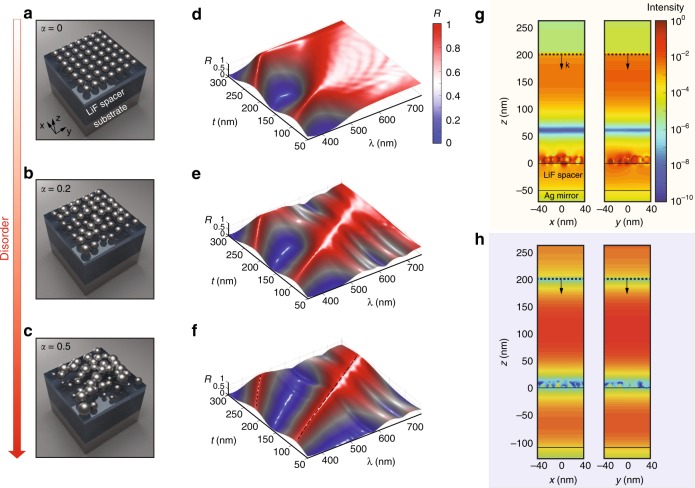
3D full-wave simulations for the disordered plasmonic system with deterministic coupling to the optical environment. **a**–**c** Schematic diagram of platforms with different disorder parameters *α* for **a** *α* = 0, **b** *α* = 0.2, **c** *α* = 0.5. **d**–**f** The corresponding reflection spectra *R* as a function of spacer thickness from FDTD simulations. **d** corresponds to setup in **a**, **e** corresponds to setup in **b** while **f** corresponds to setup in **e**. Interestingly, the introduction of disorder creates and narrows the reflection peaks. A prominent transition from broadband absorption to turnable reflection is observed in **f**. **g**, **h** Spatial distributions of the electrical field intensity in *x–z* and *y–z* plane for **g** *t* = 50 nm and **h** *t* = 110 nm. A plane wave is launched at *z* = 200 nm (blacked dashed lines) along the *y* direction with wavelength *λ* = 400 nm.

Next, we implement full-wave simulations based on the 3D FDTD method for the elucidation of the mechanism demonstrated above, which are summarised in Fig. 2. Starting from a periodic array of nanoparticles (Fig. 2a), we embed disorder into the system by introducing fluctuations to both diameters and positions of the nanospheres, represented by a parameter *α* that describes the degree of disorder (details in the Methods). Here, the thickness-dependent optical response is analysed with three setups with different values of *α* = 0, 0.2, 0.5, as depicted in Fig. 2a–c. Figure 2d–f demonstrates the corresponding simulated reflection spectra at different thicknesses respectively. When the thickness is small (*t* = 50 nm), the increasing disorder assists the plasmonic system to trap more photons inside, reducing the reflection as shown in Figs. 2d to f. For the system with intense disorder (*α* = 0.5), a reflection band is produced with increase of the thickness after a certain transition region (as shown in Fig. 2f); the spectral position of the reflection peak is controlled by the thickness. A comparison among Fig. 2d–f also clarifies the indispensable role of disorder in producing bounded reflection—only the system with broadband absorption capability can release otherwise trapped photons throughout the tunable regime. To provide more details, we illustrate intensity distributions of the electric field on *x–z* and *y–z* planes, for both the broadband absorption and tunable reflection regimes in Fig. 2g, h respectively. The size and position distribution of the nanoparticles are fixed to be the same as that of Fig. 2c, but with different spacer thickness. The situation of broadband absorption is shown in Fig. 2g while a spectrally confined reflection band is depicted in Fig. 2h. In Fig. 2g, most photons are trapped inside the plasmonic system and dissipated, with miniscule reflection (above the black dashed line), while a significant portion of the photons are reflected for the case with mismatch between 1∕*τ*_*e*_ and 1∕*τ*_*k*0_ (Fig. 2h).

### Experimental realisation

**Fig. 3:**
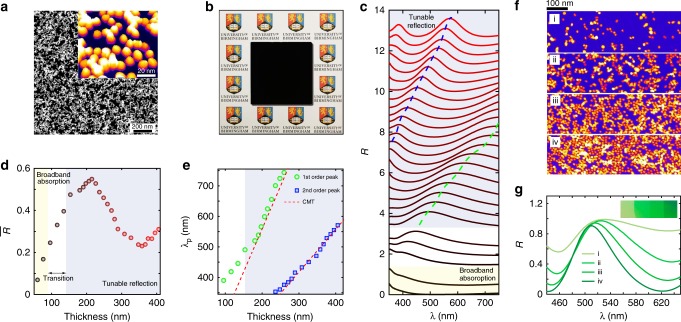
Experimental realisation of the disordered plasmonic system. **a** A typical STEM picture of Ag nanoclusters. The inset shows the 3D reconstruction plot. Disorder is introduced to the system by the deformed shapes and randomised positions of the clusters. **b** A sample with a fixed thickness around 60 nm, producing a completely black colour due to the disorder-induced broadband absorption. **c** The reflection spectra of the structure at different thicknesses, with reflection peaks marked as dashed lines. The transition is experimentally observed from broadband absorption to band-limited reflection with thickness-dependent reflection peaks. **d** The averaged reflection $\overline{R}$ from 350 to 750 nm as the thickness varies. **e** The relationship between the peak shift and thickness from experiment, which shows a good match with our model based on coupled mode theory (red dashed lines). **f** STEM pictures for the hybrid platform with four different nanoparticle densities. **g** The corresponding reflection spectra for samples with different densities. The inset shows a photographic image, illustrating the vivid colour generation from the sample with stronger disorder.

We realise the proposed system by producing Ag clusters deposited on a transparent dielectric with a gas-phase cluster beam technique^21^. A LiF spacer layer is placed on the top of a plasmonic mirror, whose thickness can be precisely controlled within nanometre precision through thermal evaporation (more details in the Methods). Figure 3a shows a typical scanning transmission electron microscope (STEM) picture of the disordered system (Fig. 3b) with a 3D reconstruction plot in the inset for more detailed morphology, demonstrating the disorder embedded in both the shape and position of the nanoclusters. Besides the direct visualisation of the randomness from Fig. 3a, we fabricate one sample with a small spacer thickness (~60 nm) in the broadband absorption regime, as shown in Fig. 3b. The experimental results indirectly demonstrate that the system carries strong enough disorder for the convergence of the decay rates *τ*_*k*0_. At the thickness with a coupling rate *τ*_*e*_ matching *τ*_*k*0_, the system is endowed with strong absorption in a broad spectral region and produces a black colour. Figure 3c summarises the reflection spectra with a gradually increased thickness of the spacer from 60 to 400 nm. With increasing of thickness, a distinct transition is observed in the spectra, as predicted by both the theoretical and numerical analyses. Starting from a small thickness, a broadband absorption is achieved by virtue of energy equipartition. After a transition region, the system enters the regime of tunable reflection, obtaining a band with red-shifted reflection peaks. Another reflection band also appears due to the second-order mode supported by the Ag film/spacer/Ag clusters structure. Figure 3d shows the averaged reflection $\overline{R}$ in the visible, with corresponding thickness illustrated for different spectra in Fig. 3c. The transition from broadband absorption to tunable reflection is verified by the value of $\overline{R}$, from a monotonical increase to oscillation. The relationship between reflection peak *λ*_p_ and thickness of the dielectric layer is plotted in Fig. 3e. According to our theory, the mode confined in the Ag film/spacer/Ag clusters would not be absorbed but released, with its reflection peak located at *λ*_p,CMT_ = 2*n*_s_*t*∕*m*, with *n*_s_ the refractive index of its spacer, *t* the thickness and *m* the order of the mode.

To clarify the indispensable role of disorder for the proposed system experimentally, we fabricated systems with different nanocluster density, as illustrated in Fig. 3f, with coverage rates of 8.5%, 30.8%, 45.2% and 51.8%. When the density is low, the interactions among the clusters are negligable (as the coupling strength between the nanoparticle decays strongly with the increase of the distance), only the disorder in shape plays a dominant role. As the density increases, the interaction (near-field coupling) strength grows nonlinearly, intensifying the disorder embedded in the system. Figure 3g plots the reflection spectra of the samples with three different densities. Note that the amplitude of the reflection spectra is normalised to exclude the effect of absorption variation resulted from the increment of the density of plasmonic nanoclusters. The bandwidth shrinks with increasing density (disorder), agreeing with the result shown in Fig. 2d–f. The inset shows a picture of the samples with colour shining more brilliantly as the density increases. This visually shows the beneficial role that disorder plays.

**Fig. 4:**
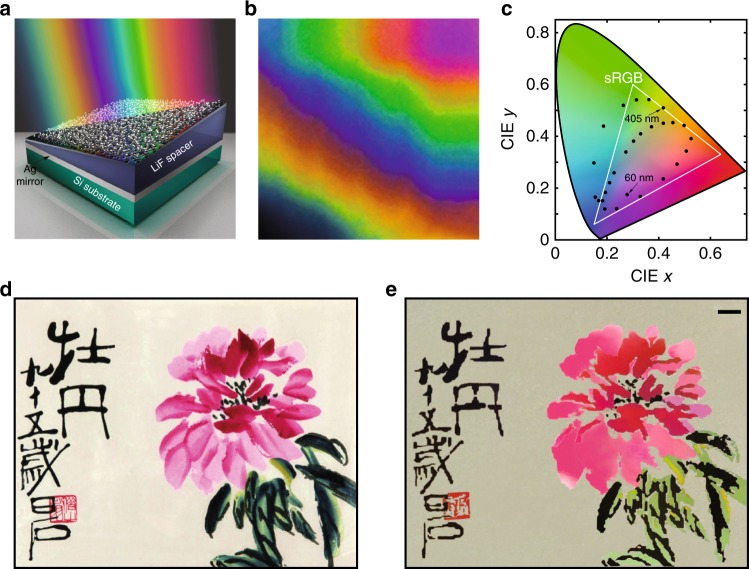
The versatility of the disordered plasmonic system for colouration. **a** Schematic of the sample with Ag nanoclusters on a spacer with gradually varying thickness along the diagonal. **b** Photographic image of the structure depicted in **a**, with rainbow-like colours starting from black along the diagonal where coupling strength is tuned. **c** The diverse colour generation in CIE 1931 xy chromaticity diagram with black dots calculated from the spectra from Fig. 3c and the sRGB gamut outlined in white lines. **d** Digital copy of the painting (Peony Flower). **e** The fabricated hybrid structure. The scale bar represents 10  mm.

To demonstrate the ability for continuous tuning, we fabricate a sample with a linearly increased spacer thickness along its diagonal (as depicted in Fig. 4a). Figure 4b provides a photographic image of the sample. After a transition from black at the bottom left corner, we observe a rainbow-like colour variation along the diagonal, demonstrating the high tunability of the reflection peaks. Naturally, such a platform can be used as a novel mechanism for structural colour generation^22^. Compared with the conventional methodology utilising a deterministic resonant structure to filter (absorb) unwanted light, our spacer thickness here is designed to release photons with a desired colour. From a practical point view, taming the disordered system formed by merely a single step of deposition brings a crucial benefit: it relieves the fabrication from sophisticated and/or time-consuming techniques (such as top-down process based on lithography) required for periodic nanostructures^22–24^. This fact is especially beneficial for colour decoration at large scales. In Fig. 4c, we plot the International Commission on Illumination (CIE) 1931 xy chromaticity diagram, with corresponding chromaticity calculated and marked (black dots) from the reflection spectra in Fig. 3c (see Supplementary Note 8 for more details). The colours have a broad range that can cover most of the standard Red Green Blue (sRGB) gamut (inside the white triangle), providing enticing prospects for colour displays. Quite interestingly, disorder endows the hybrid plasmonic system with a unique feature: randomness inside the system can counterintuitively enhance the resonance controllability (colour coverage). Compared with the CIE diagram from the simulation (Supplementary Note 7), the experimental results outperform the simulated ones, due to stronger disorder generated experimentally from fabrication imperfection, while in the numerical study, the nanoparticles are treated as perfect spheres with different radii for the simplicity of simulation and control of disorder. Further increment of the level of disorder in numerical calculation can enlarge the coverage in the CIE diagram (Supplementary Note 9). However, the intrinsic imperfection in the fabrication endows the realistic structures a better colour availability.

To highlight the feasibility of colourful pattern generation, we print the Chinese watercolour painting “The Peony Flower” by Baishi Qi, as shown in Fig. 4d–e. The original work is presented in Fig. 4d, containing several ubiquitously used colours such as red, pink, yellow and green. Meanwhile, it also requires black colour (for Chinese calligraphy) that can be easily achieved with our system working in the broadband absorption regime; we note that generating black remains as a formidable task for conventional periodic structures. In our system, all desired colours including black can be formed by simply tuning the spacer thickness to replicate the original work, as shown in Fig. 4e. Coloration at oblique angles is also investigated in Supplementary Note 11.

## Discussion

While the broadband absorption feature of disordered systems is well studied and judiciously utilised, in this letter, we shed light on a disordered system, unveiling a novel regime with band-limited properties with excellent tunablity. Such an extending of understanding of disordered systems could initiate a new pathway for designs in disordered photonics^25^. A theory is developed based on a generalised model (CMT), which can be applied to different optical or even microwave disordered systems other than the plasmonic structures. While we introduce the rate mismatch by decreasing the intrinsic decay rates in the current configuration, the coupling efficiency *η* can also be reduced by increasing the intrinsic loss (as shown in Fig. 1c). Consequently, similar phenomena may be achieved by enhancing the loss for specific modes, for example, through Purcell effects from disordered systems embedded inside a cavity.

Apart from its theoretical importance, the mechanism elucidated here can be feasibly applied to different applications. In the show-of-concept example shown above, we circumvent the inevitable but undesired fabrication imperfection, leading to a crucial benefit of vivid colour generation. Considering the outstanding sensitivity of the generated colour to the spacer thickness (as shown in Fig. 3d), our hybrid system may allow on the realisation of backlight-free dynamical colour displays, utilising advantages of recently-developed nanoelectromechanical systems^20,26,27^. Compared with traditional methods to tune the reflection spectrum by resonant structures such as Fabry Perot to absorb unwanted colour in the spectrum^22–24,28^, we provide a novel path to utilise a transparent (lossless) cavity to directly release photons in the mode it supports (see more details in the Supplementary Note 4). Not limited to colour generation, tunable colour variation can be utilised as a visualisation of external stimuli, such as mechanical pressure or acoustic waves using a flexible spacer.

Here we applied our mechanism to generate structural colour as one demonstration; however, the ability to manipulate disordered systems suggests a broader set of applications. Compared to conventional disordered cavities, the top plasmonic nanoparticles provide intensive field enhancement for improved fluorescence or photon-current generation. Besides, the plasmonic enhancement can be selectively shielded by an external cavity (as shown in Fig. 2g, h).

Compared with metal–insulator–metal heterostructure that successfully generated vivid colours^29–31^, our configuration unveils interesting properties of disordered systems, which are traditionally believed to be broadband. Also, material-independent structural colour can be realised by utilising the power of disorder, considering that the random network is not necessarily composed of Ag but any plasmonic materials.

## Methods

### 3D FDTD full-wave simulations

All the FDTD simulations are based on a commercialised software (LUMERICAL, FDTD Solution). The refractive index of Ag is based on the data from ref. ^32^ while the refractive index of LiF is from ref. ^33^. For the periodic array of Ag nanoparticles, we choose the diameter of *d*_0_ = 10 nm, with 7 × 7 NPs form the networks. The distance between each NP is 2 nm. The disorder is introduced to the system by the fluctuations of the diameters *d* and positions (*x*^*i*^, *y*^*i*^) of the NPs,

2 $$ d={d}_{0}\left[\right.1+\alpha {U}_{1}(x,y)\left]\right.,$$

3 $${x}^{i}={x}_{0}^{i}+(\alpha * {d}_{0}){U}_{2}(x,y),$$

4 $${y}^{i}={y}_{0}^{i}+(\alpha * {d}_{0}){U}_{3}(x,y),$$

with (${x}_{0}^{i}$, ${y}_{0}^{i}$) the position of the NPs in the periodic array, *d*_0_ the original diameter, *U*_1_, *U*_2_, *U*_3_ three independent uniform distributions in [−1, 1]. *α* is a control parameter to represent the degree of disorder inside the system, as demonstrated in Fig. 2a–c in the main text. Figure 2a–c illustrates the trend as the increment of the position disorder embedded in the photonic system. The simulation domain is less than 100 nm in *x* and *y* direction to demonstrate the phenomenon, indicating the potential of the colour pixels at sub-wavelength scale.

The system containing disorder only in position or shape is investigated in Supplementary Note 2. To exclude the effect from a specific reconfiguration, we implement the simulations with another three different sets of random seeds and the results are similar (as shown in Supplementary Note 3).

### Sample fabrication

Silver (Ag) and lithium fluoride (LiF) films were deposited in a thermal evaporation system (SinoRaybo Nanoscience and Nanotech Co., Ltd). All evaporation were performed at a high vacuum, the working pressure of the system is about 1.7 × 10^−7^ Torr. The deposition rate and film thickness were monitored by a quartz crystal microbalance. The LiF film with different thickness presented in the work were all prepared by choosing a suitable substrate position on the substrate holder and movement during thermal evaporation through a fixed shutter (see Supplementary Note 5 for more details). Gas-phase cluster beam deposition process was used to deposit Ag clusters on the surface of Ag film/spacer layer structure with details demonstrated in Supplementary Note 5. The details of fabrication of the watercolour painting by Baishi Qi can be found in Supplementary Note 10.

### Sample characterisations

The STEM investigation was performed using a JEOL instrument (JEM2100F) with a spherical-aberration corrector (CEOS GmbH). The images were acquired using high-angle annular dark field and bright field detectors. The thickness of the spacer was characterised by scanning electron microscopy (Hitachi S4800). The reflection spectra of the samples were measured by a Zolix *λ* 300 Spectrograph & Monochromator. See Supplementary Note 6 for more details.

## Supplementary information

Supplementary Information

## Data Availability

All the data supporting the findings of this study are available within the article, its Supplementary Note files, or from the corresponding author upon reasonable request.

## Code availability

Upon reasonable request, the authors will provide related code for the purpose to reproduce the results of this paper.
