# Supplementary material for: Manipulating disordered plasmonic systems by external cavity with transition from broadband absorption to reconfigurable reflection
Source: Nat Commun. 2020 Mar 24;11:1538. doi: 10.1038/s41467-020-15349-y (PMC7093388; doi:10.1038/s41467-020-15349-y)
Supplement: Supplementary file 1 — Supplementary Information [file 41467_2020_15349_MOESM1_ESM.pdf]

Supplementary Materials for "Manipulating disordered plasmonic systems by external cavity with transition from broadband absorption to reconfigurable reflection"

Peng Mao,[1,2,5] Changxu Liu,[1,3,5], Fengqi Song[4], Min Han,[4*],
Stefan A. Maier[3*], Shuang Zhang[1*]

[1]School of Physics and Astronomy, University of Birmingham, B15 2TT, United Kingdom,
[2]College of Electronic and Optical Engineering & College of Microelectronics,
Nanjing University of Posts and Telecommunications, Nanjing 210023, China
[3]Chair in Hybrid Nanosystems, Nanoinstitute Munich, Faculty of Physics,
Ludwig Maximilian University of Munich, 80539 Munich, Germany
[4]National Laboratory of Solid State Microstructures, Collaborative Innovation
Center of Advanced Microstructures, and College of Engineering and Applied Sciences,
Nanjing University, Nanjing,210093, China
[5]These authors contribute equally to this work
[*]To whom correspondence should be addressed; E-mail: sjhanmin@nju.edu.cn
stefan.maier@physik.uni-muenchen.de s.zhang@bham.ac.uk.

Supplementary Note 1: more details of system modelling based on coupled mode theory

The modes inside the optical cavity can be expressed as:

$$a_k(t) = A_k e^{i\omega_k t - t/\tau_k} \tag{1}$$

with ω_k the frequency, $1/\tau_k$ the decay rate (inverse lifetime) considering both the coupling and intrinsic loss $1/\tau_k = 1/\tau_{k0} + 1/\tau_e$. $\mathcal{E}_k = |a_k|^2$ is the energy store in the kth mode. It obeys the relationship as the following (*1*):

$$\frac{\mathrm{d}a_k}{\mathrm{d}t} = \big(i\omega_k - 1/\tau_k\big)a_k + \sqrt{\frac{1}{\tau_e}} \cdot S(t) \tag{2}$$

with $S(t)$ the input pump. While the reflection R_k and transmission T_k for kth mode obeys (*1*):

$$R_k = -\sqrt{\frac{1}{\tau_e}} a_k \tag{3}$$

$$T_k = -\sqrt{\frac{1}{\tau_e}} a_k + S \tag{4}$$

For a CW pump $S(t) = e^{i\omega t}$, the a_k can be solved as:

$$a_k = \sqrt{\frac{1}{\tau_e}} \frac{e^{i\omega t} - e^{i\omega_k t - t/\tau_k}}{i(\omega - \omega_k) + 1/\tau_k} \tag{5}$$

For the total energy $\mathcal{H}$ stored in the cavity can be derived from equation (5):

$$\mathcal{H} = \int \frac{\mathrm{d}\omega}{\tau_e} \sum_k \frac{1 + e^{-2t/\tau_k} - 2\cos\big[(\omega_k - \omega)t\big] e^{-t/\tau_k}}{(\omega_k - \omega)^2 + 1/{\tau_k}^2} \tag{6}$$

Considering the integral yields significant contributions only for $\omega \approx \omega_k$, the equation can be simplified to:

$$\mathcal{H} \approx \frac{1}{\tau_e} \sum_k \tau_k^2 (1 - e^{-t/\tau_k})^2 \Delta\omega \tag{7}$$

The power $P_k = \frac{\partial |a_k|^2}{\partial t}$ transferred into the kth mode can be also expressed as (from energy conservation):

$$P_k = |S|^2 - |R_k|^2 - |T_k|^2 \tag{8}$$

Combined with equations (3,4), the absorbance of the mode (coupling efficiency) $\eta = P_k/|S|^2$ can be expressed as:

$$\eta = \frac{2\tau_e/\tau_{k0}}{(1+\tau_e/\tau_{k0})^2} \tag{9}$$

.

When the system attaches to an external cavity which supports specific modes ω_j, the corresponding decay rate $1/\tau_{j0}$ is perturbed. The confinement of mode ω_j in the external cavity mitigates the light-matter interaction in the original system, reducing the decay rate $1/\tau_{j0}$ for the setup shown in the lower panel of Fig.1d in the main text. Despite the difficulties in writing an analytical solution of the variation of the rate (due to disorder), the decay rate qualitatively decreases, causing the mismatch to τ_e. Consequently, the mode ω_j supported by the cavity is released outside (absorbance η reduces).

Supplementary Note 2: analysis for different types of disorder

In the main text, we analysis the case for the disorder in both size and position of the Ag clusters, to match the experimental configurations. Here we also implement simulations with either disorder in the size or the position to clarify the contributions from different types of disorder. The results are summerized in Fig. S1. Similarly, The disorder in either shape or position may lead to the reflection band formations (Fig. S1C-D) at different thickness. However, the reflection bandwidth is increased for single type of disorder, which also verified by the reduced absorption A = 1- R at t=50nm according the mechanism we discussed in the main text.

This phenomenon verifies that the disorder level determines the sharpness of the reflection band, as demonstrated in the main text. In the fabrication process, more complicated disorder in shape and position is intrinsically induced, driving the system to a strong chaotic regime which is desired here.

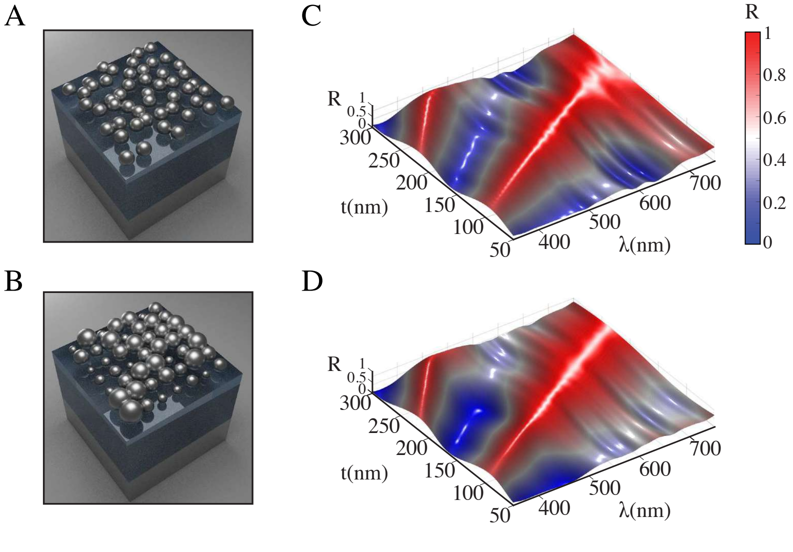

Supplementary Figure 1: Comparison with different disorder tyeps. (A-B) The schematic illustrations of the disordered system with disordered only embedded in position A) and size B) with $\alpha = 0.5$. (C-D) The corresponding reflective spectra as a function of thickness.

Supplementary Note 3: simulations with different random seeds

To show the reflection tunability is a general effect resulted from the interaction between external cavity and the disordered plasmonic system other than a specific arrangement, we implement additional simulations with different random seeds (U_1, U_2 and U_3). Figure S2 summarises the reflection spectra with three different sets of random seeds, with similar results compared with Fig.2F in the main text.

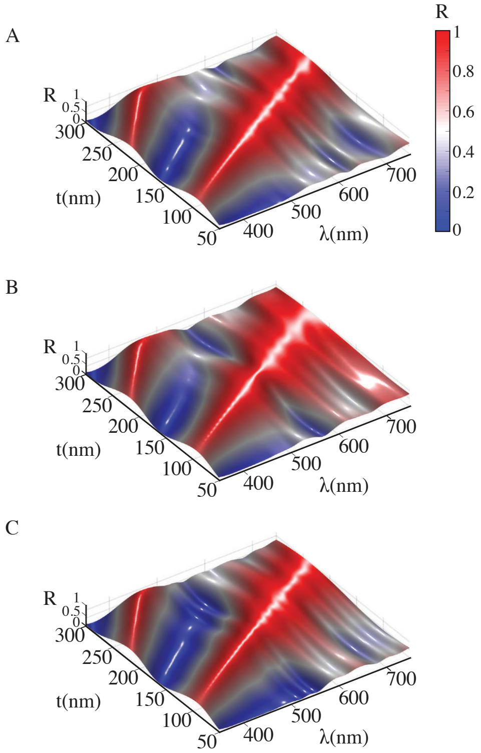

Supplementary Figure 2: Comparison of the result from Fig.2F in the main text with different sets of random seeds U_1, U_2 and U_3 (in equations(2-4) in the main text).

Supplementary Note 4: indispensable role of both disorder system and external cavity to achieve good tunability

To demonstrate the tunability is achieved with both disorder system and the external cavity (Fig. S3A-B), we implement additional simulations as the control groups, which is summarised in Fig. S3.

Fig. S3A-B directly copy the results from Fig.2C and F in the main text as a reference. In Fig. S3C, we replace the disordered plasmonic system with a silver film with the same averaged thickness, turning the structure into a Fabry-Perot resonator. The reflective spectra are demonstrated in In Fig. S3D, with reflection dips instead of reflection peaks. The difference elucidates the working principle of our system - the external cavity assists the corresponding mode to release (not be absorbed) outside the system owing to the lifetime mismatch while traditional cavity enhances the absorption of the mode. For a more straightforward comparison, we map the two sets of spectra into the CIE xy chromaticity diagram, as shown in Fig. S4. Since the feasibility to select one spectrum band to generate a specific colour (than removing all the unwanted wavelength in the visible spectrum), the disordered system with an external cavity achieves a broader colour range than a Fabry-Perot resonator. By using the configuration we proposed, it readily produces vivid green colour by selecting the thickness of the spacer (Fig. 4B in the main text), while the absorption-based systems suffer absorbing red and blue colour simultaneously (*2–4*).

Fig. S3E investigates the situation when the plasmonic mirror on the backside is removed, drastically reducing the light confinement of the cavity. In addtion, the impinging light does not experience strong enough interactions with the ultra-thin plasmonic structures to "see" the disorder, leading to small reflection shown in Fig. S3F.

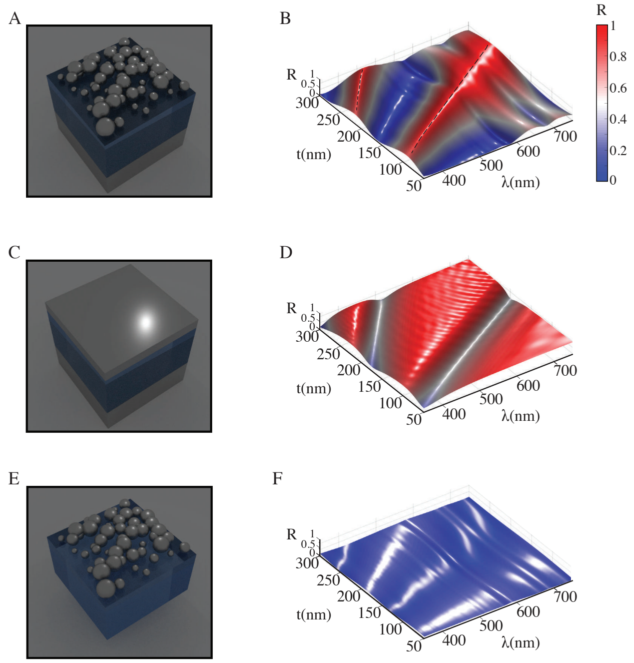

Supplementary Figure 3: (A-B) Disordered plasmonic system with an external cavity and the corresponding reflection spectra. A copy from Fig. 2e-f as a reference. (C-D) A Fabry-Perot resonator formed by replacing disordered system in A with a thin film and the corresponding reflection spectra. (E-F) Disordered plasmonic system with an external cavity but without the plasmonic mirror on the back and the corresponding reflection spectra.

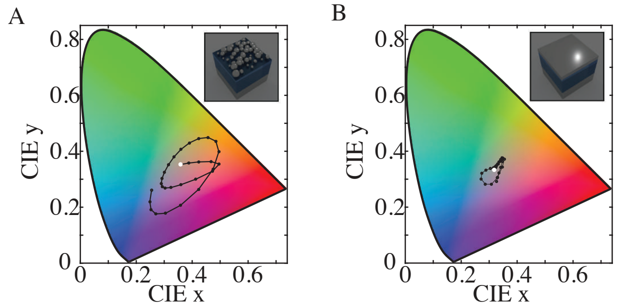

Supplementary Figure 4: The CIE diagramed mapped by the reflection spectra from (A) Disordered plasmonic system with an external cavity and (B) A Fabry-Perot resonator. The white dot is starting point with thickness of 50nm.

To further demonstrate the role of the plasmonic mirror, we illustrate the spatial distribution of the electric field for two structures - one with the mirror and another without it, as illustrated in Fig. S5. From a comparison between the case with plasmonic nanoclusters (left panel in both Fig. S5A and B) and the case without plasmonic nanoclusters (right panel in both Fig. S5 A and B), the level of light-matter interaction can be illustrated. The relative reflection attenuation with incorporation of the plasmonic nanoclusters demonstrates the absorption of the disordered system, while the position variation of the peaks or dips of the electric field demonstrates the induced phase shift. An enhanced absorption of the structure with mirror (Fig. S5 A) can be observed, compared with the structure without mirror (Fig. S5 B). The results match the data shown in Fig. S3F and Fig. 2f in the main text. A clear phase shift in Fig. S5A further confirms the strong light-matter interaction, which is in sharp contrast with the minute variation in Fig. S5B.

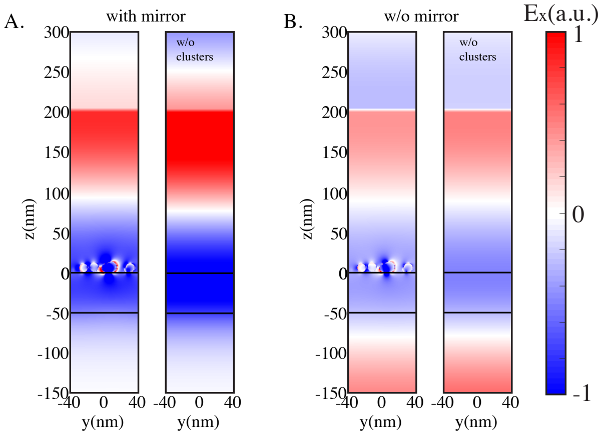

Supplementary Figure 5: Electric field distributions of disordered plasmonic network (A) with (B) without a plasmonic mirror on the backside. A reference without plasmonic network is also demonstrated on the right for each panel.

Supplementary Note 5: sample fabrication

Thermal evaporation of Ag mirror and LiF spacer

The LiF film with different thickness presented in the work were all prepared by choosing a suitable substrate position on the substrate holder and the movement during thermal evaporation through a fixed shutter, as schematically shown in Fig. S6. During the fabrication of the spacer with varying thickness (Fig. 1a in the main text), a stepper motor was equipped on the evaporator chamber to control the horizontal movement of the substrate with respect to the evaporated flux. The fixed shutter has a square opening that only allows a section of the sample to be exposed to the evaporated materials at any given time. As the sample is constantly moving during the evaporation with a speed of v, the exposed section of the sample is decreasing, resulting in a gradient morphology of the film, as schematically shown in Fig. S6.

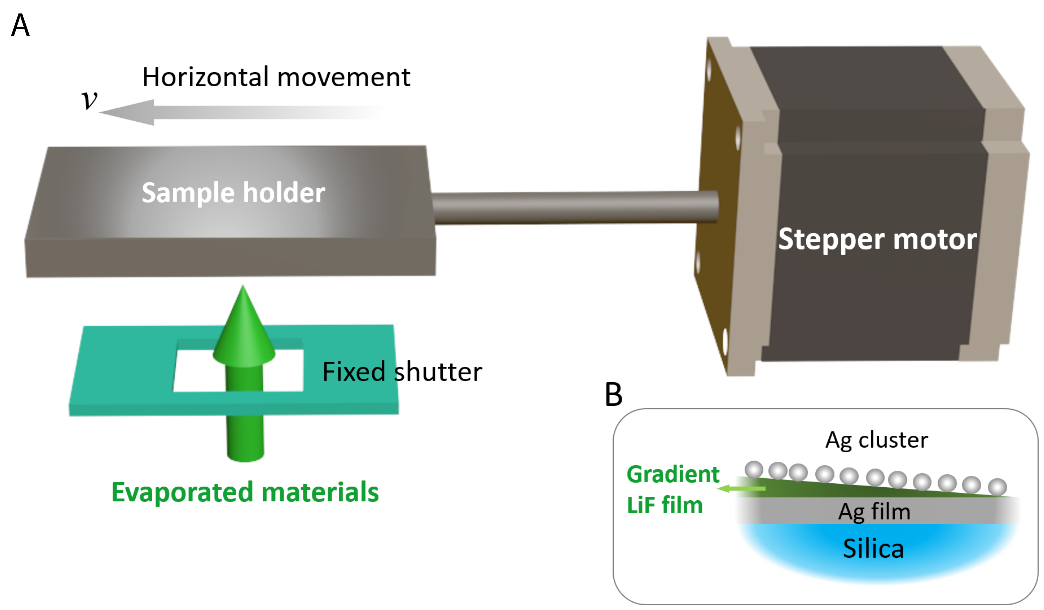

Supplementary Figure 6: (A) Schematic showing the evaporation apparatus used to prepare gradient film. (B) Schematic of Ag film/LiF film/Ag clusters nanostructure with the thickness of the LiF spacer continuously increasing.

Gas-Phase Ag Cluster beam deposition

Gas-phase cluster beam deposition process was used to deposit Ag clusters on the surface of Ag film/spacer layer structure (*5*). In this fabrication, Ag clusters were generated in a magnetron plasma gas aggregation cluster source and deposited on substrates directly, as shown in Fig. S7A. The deposition was performed in a high-vacuum chamber equipped with the cluster source. A silver plate (50mm·3mm) with high purity (99.99%) was used as the sputtering target. A DC power supply was used for the sputtering of Ag target in argon gas (purity, 99.99%) ambient with a pressure of $\sim$100 Pa, maintained by passing argon gas to the liquid nitrogen-cooled aggregation tube. A stable magnetron discharge ran with input power of 40 W. Pure Ag clusters were initially formed through a supersaturated vapour of metal atoms by sputtering of the target under argon atmosphere. The clusters were swept by the gas stream out of the aggregation tube into vacuum through a diaphragm, where the clusters growth was effectively stopped. The clusters continued to pass through a skimmer (the second diaphragm) into a high vacuum (10^{-4} Pa) chamber and forming a collimated nanoparticle beam with a high speed of $\sim$1000 m/s, and then deposited on the surface of substrates. The cluster beam deposition rate was monitored by a quartz crystal microbalance (QCM). During the deposition, the cluster beam can be cut off by a shutter instantly to make a careful control of the deposition coverage.

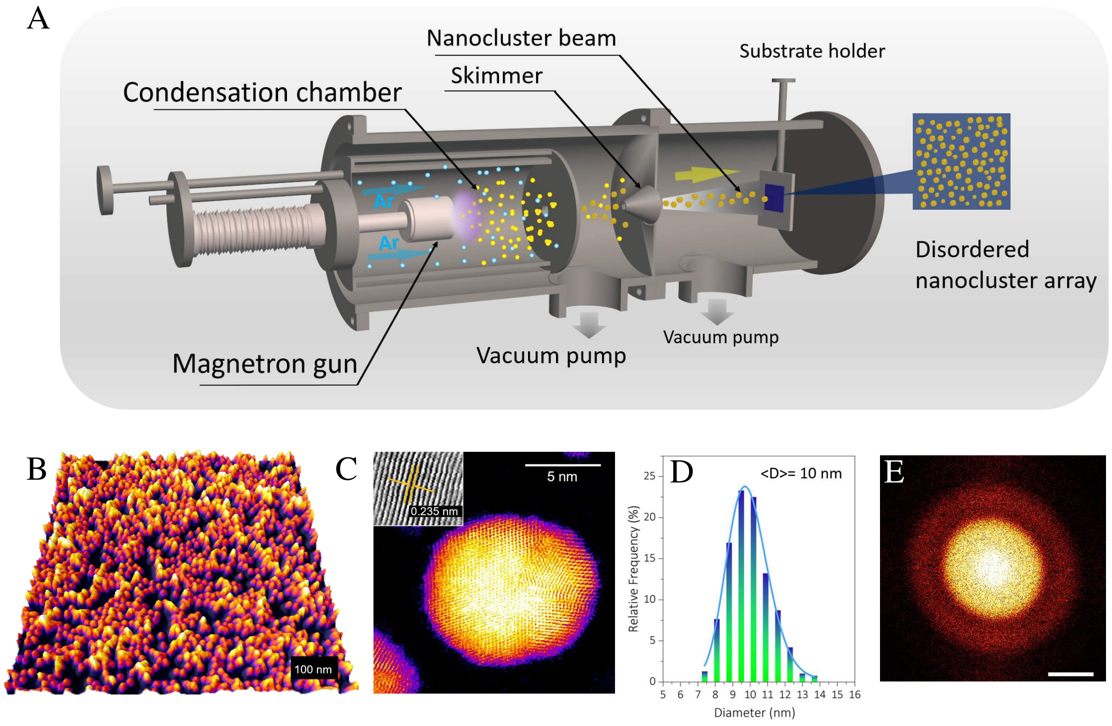

Supplementary Figure 7: (A) Schematic diagram of gas-phase cluster beam deposition system. (B) 3D-plot HAADF-STEM image of Ag cluster array. (C) Typical HAADF-STEM image a single Ag cluster. The inset is BF-STEM image of Ag cluster and the corresponding lattice constant has been marked in the figure. (D) Size distribution of as-deposited Ag clusters. (E) 2D-FFT of the positions of the Ag nanoclusters, which is calculated from the STEM image of disordered Ag system. Scale bar corresponds to 200 μm^{-1}.

Supplementary Note 6: sample characterizations

STEM characterizations of the Ag clusters

Figure S7B shows the HAADF-STEM image as-deposited Ag clusters. As seen, Ag clusters are randomly distributed on the substrate and form numerous closely spaced cluster-assembling areas. These randomly distributed Ag clusters on the substrate resulted in the formation a disordered plasmonic system. Figure S7C shows the HAADF-STEM and BF-STEM images of a single Ag cluster. The lattice fringes have an inter-planar spacing of 0.235 nm, corresponding to the (111) planes of the face-centered cubic (FCC) structure of metallic Ag, which clearly reveals the crystalline nature of the Ag clusters synthesized in the present study. The statistics of the size distribution from the gas-phase cluster beam deposition system is demonstrated in Fig. S7D. The average size of as-deposited Ag clusters is about 10 nm.

For the further illustration of the disorder, we performed the two-dimensional Fast Fourier Transform (2D-FFT) based on the positions of Ag nanoclusters (from the top view STEM image) in Fig.S7E. A circle-shaped distribution of the squared Fourier components is observed, confirming the disordered arrangement of Ag nanoclusters (*6, 7*).

SEM characterizations of the hybrid system

The structure of the Ag film/LiF/Ag cluster sample was characterized by scanning electron microscopy (SEM, Hitachi S4800). Figure S8A show the optical photographs of blank silica, Ag film/LiF sample and Ag film/LiF (60nm)/Ag cluster sample. Figure S8B show the optical photographs and SEM images of different colour Ag film/LiF/Ag cluster structures, the thickness of LiF film has been marked in the cross-sectional SEM images. Such 8 colors can be feasibly used as the a 3-bit information storage system.

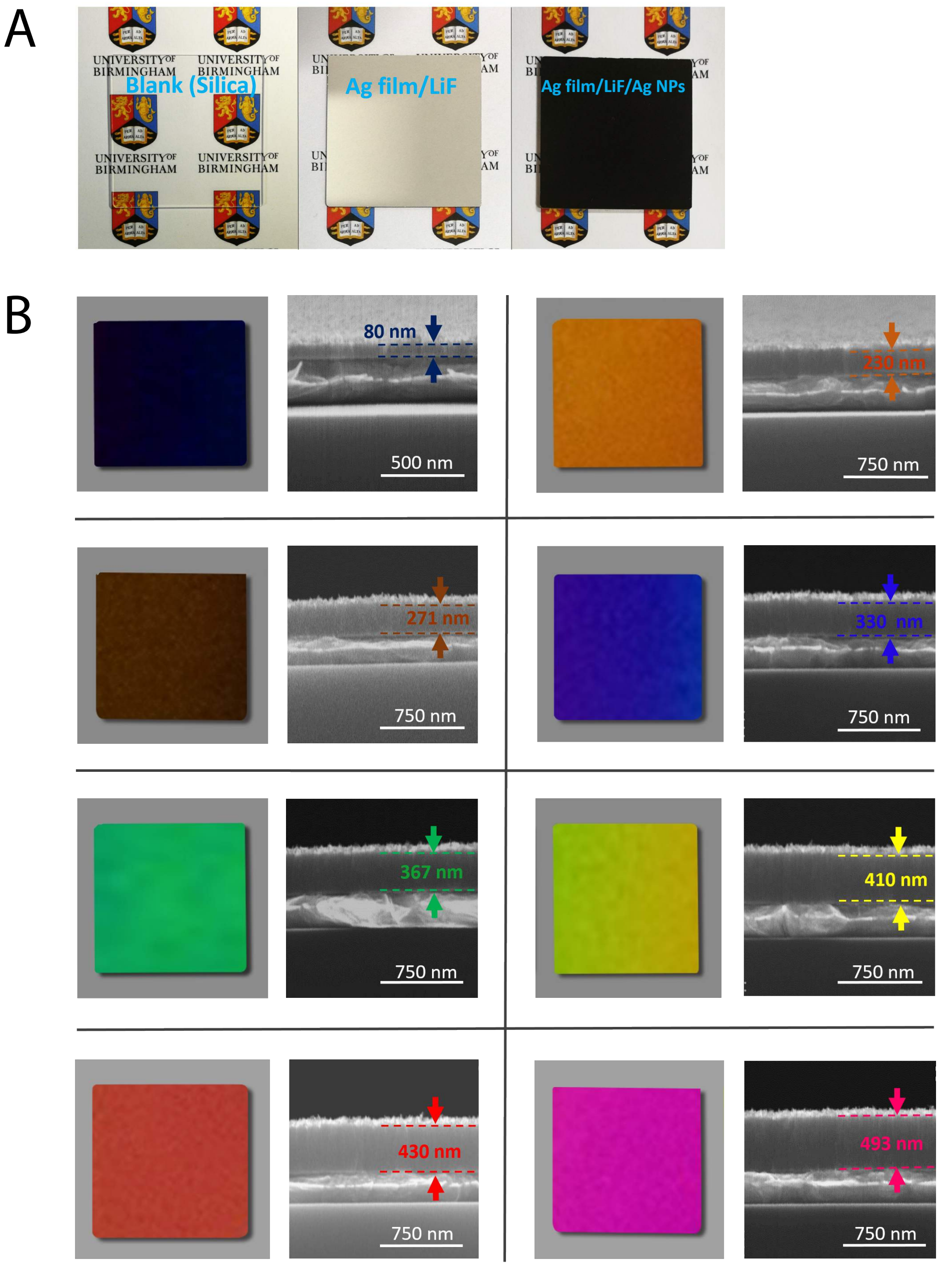

Supplementary Figure 8: (A) Optical photographs of blank silica, Ag film/LiF sample and Ag film/LiF/Ag clusters sample. (B) Optical photographs and SEM images of the samples with different colour. The difference between these samples is only the thickness of LiF film. The thickness of LiF film has been marked in the cross-sectional SEM images.

Supplementary Note 7: quantitative colour analysis based on CIE chromaticity diagram

To quantitative clarify the colour range that our system can cover, we project the reflection spectra into the Commission on Illumination (CIE) 1931 xy chromaticity diagram ((more details can be found in the following section)), as summarised in Fig. S9. Simulated results with different disorder parameter α while experimental results are duplicated from the main text as a reference. For the simulated spectra in Fig. S9A-C, more colour area is achieved as the disorder embeded in the system increase, matching the reflection band shirking described in the main text.

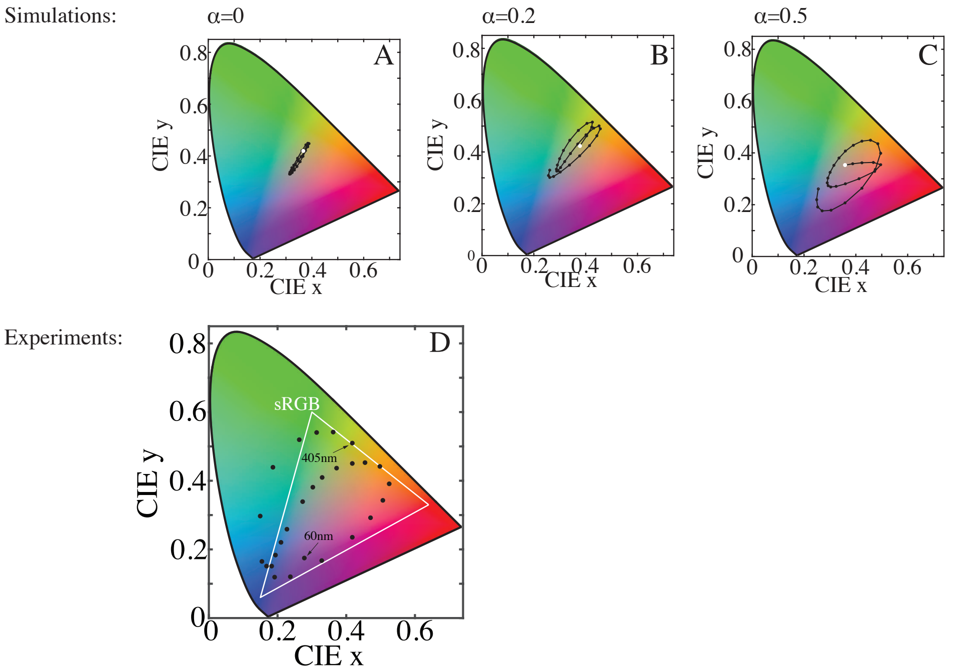

Supplementary Figure 9: Color projection to CIE 94 diagram from simulated and experimental reflection spectra. (A-C) Simulated data with different disorder A)$\alpha = 0$, B)$\alpha = 0.2$, C)$\alpha = 0.5$. (D) Experimental data.

Supplementary Note 8: details of color projection to CIE chromaticity diagram

According to The International Commission on Illumination (CIE), there is a standard method of specifying the color of illuminants or materials by tristimulus values X, Y , Z (*8*):

$$X = K \int P(\lambda)\overline{x}(\lambda)\mathrm{d}\lambda \tag{10}$$

$$Y = K \int P(\lambda)\overline{y}(\lambda)\mathrm{d}\lambda \tag{11}$$

$$Z = K \int P(\lambda)\overline{z}(\lambda)\mathrm{d}\lambda \tag{12}$$

with $P(\lambda)$ the is spectral power, K a scaling factor. $\overline{x}(\lambda)$, $\overline{y}(\lambda)$, $\overline{z}(\lambda)$ color-matching functions (CMFs) correspond to three kinds of cone cells of human eyes in short ("S", 420nm - 440nm), middle ("M", 530nm - 540nm), and long ("L", 560nm - 580nm) wavelengths. For the reflective colors, the spectral power can be decomposed to into two terms $P(\lambda) = S(\lambda) \cdot R(\lambda)$, where $R(\lambda)$ is the reflectivity and $S(\lambda)$ is the relative power of the illuminant shining on the system.

The tristimulus values X, Y , Z are normalized to CIE xy color space, with x,y containing the only chromaticity information (brightness is missing due to normalization):

$$x = X/(X + Y + Z) \tag{13}$$

$$y = Y/(X + Y + Z) \tag{14}$$

$$z = Z/(X + Y + Z) = 1 - x - y \tag{15}$$

Here, we choose color-matching functions as the CIE 1931 2° Standard Observer Observer function, as demonstrated in Fig. S10A. For the illuminant in the above section, we select the

CIE Standard Illuminant D65 as $S(\lambda)$ (*9*), which corresponds to average daylight and has a correlated colour temperature of approximately 6500 K (as shown in Fig. S10B). We also calculate the CIE xy chromaticity diagram with a different $S(\lambda)$ (*9*), that is Illuminant A representing typical, domestic, tungsten-filament lighting, as plotted in in Fig. S10B. The results are demonstrated in Fig. S10C, with a color shift to red compared to Fig.1h in the main text. All the images in the main text are took under the fluorescent cubes in the lab, which may have some difference with the standard illuminant.

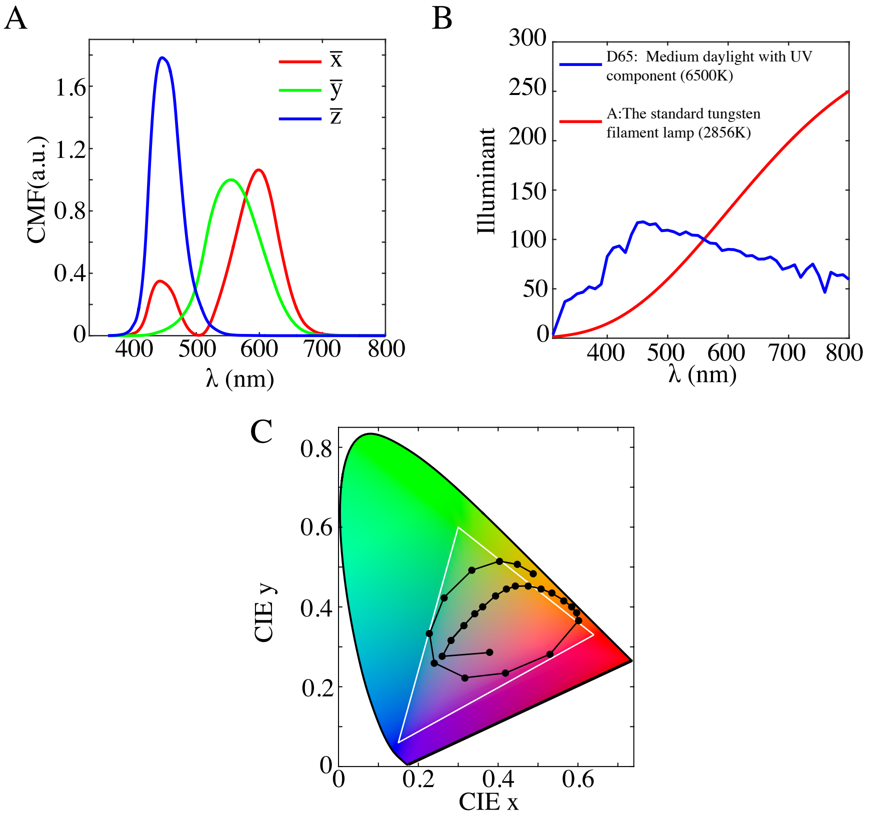

Supplementary Figure 10: (A) CIE 1931 2° Standard Observer Observer functions. (B) CIE standard illuminant Illuminant D65 and Illuminant A. (C) CIE 1931 xy space at a Illuminant A for the experimental results shown in Fig. 1H.

Supplementary Note 9: an improved match for experimental results

In the main text, we demonstrate the trend that the reflection band narrows when the disorder increases. To demonstrate the mechanism, we simply model the nanoparticles as a perfect sphere and assume that the disorder level in shape and position is the same. Figure S11 demonstrates the absorption spectra around critical coupling for the numerical results (with different values of α) and the experimental one. Despite the absorption increases with disorder (α) in simulations, it cannot compete with the experimental results with stronger disorder embedded (*10*). Such lack of absorption leads to smaller colour coverage in simulations, as illustrated in Fig. S9.

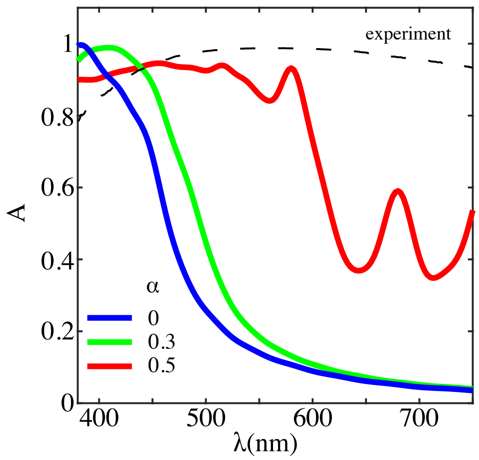

Supplementary Figure 11: A comparison of absorption around critical coupling (thickness t=60 nm) for simulations (with different values of α) and experimental results (black dashed line).

Here we introduce more disorder to the simulation, i.e., treating the nanoparticle as an ellipsoid instead of a sphere. Now there are three degrees of freedom - lengths of the three principal axes instead of the diameter of the sphere. The length of in x axis is defined as $d_x = d_0[1 + \alpha_x^s U_x]$. And the rest of two lengths (in y and z) is $d_{y,z} = d_x[1 + \alpha_{y,z}^s U_{y,z}]$. Again, $U_{x,y,z}$ is independent uniform distribution in [-1, 1] while $\alpha_{x,y,z}^s$ is a control parameter for the

disorder in shape. α_i^p is another control parameter for the disorder in position as defined in eq. (3-4) in the main text. Figure S12 summarised the numerical results for a disordered plasmonic network with $\alpha^s_{x,y,z} = 0.25$ and $\alpha_i^p = 0.3$. The schematic of the platform is shown in the inset of Figure S12A, demonstrating the deformation from spheres to ellipsoids that introduces disorder in the other two degrees. The enhancement of the level of disorder is verified by the absorption spectrum around the critical coupling (t=60nm), as shown in Fig. S12A. The reflection spectra and the corresponding CIE diagram is shown in Fig. S12B and C, separately. An improved match to experimental data (Fig. 3 and Fig. 4) is achieved owing to the disorder enhancement.

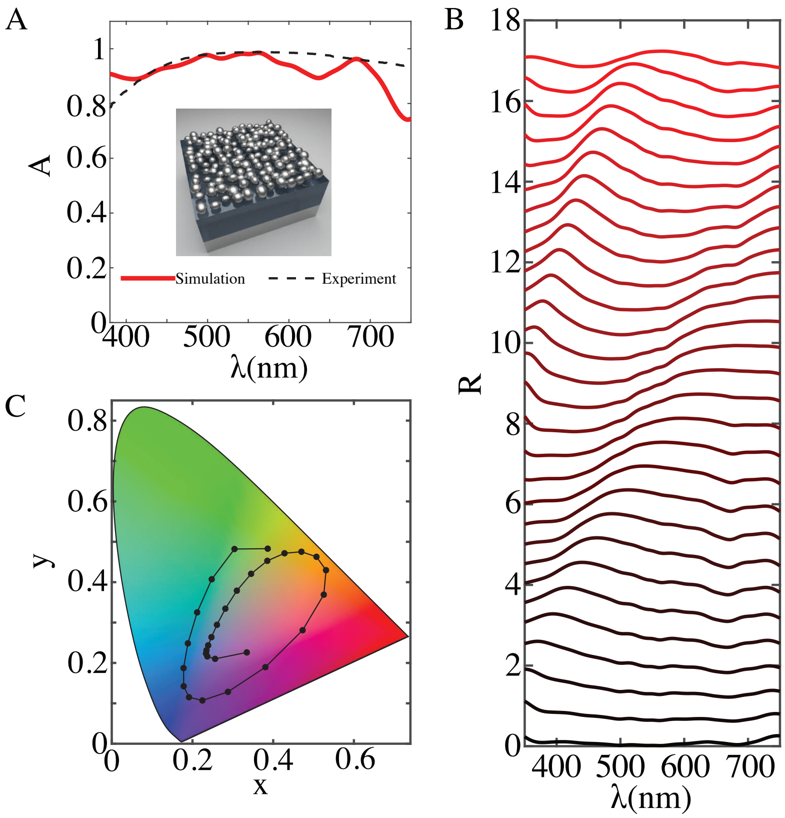

Supplementary Figure 12: (A) The comparison of absorption around critical coupling (thickness t=60 nm) between simulation (red solid line) and experimental results (black dashed line). (B) The reflection spectra from simulations for different values of t. The thickness t varies from 60 nm to 340 nm, with an interval of 10nm. (C) The corresponding CIE 1931 xy chromaticity diagram calculated from B).

Supplementary Note 10: fabrication of the watercolor painting by Baishi Qi

The pixel-based painting is obtained and the colors and size of the pixel is further discretized. The bottom Ag layer of ∼200 nm was deposited by the thermal evaporation on the top of a silica substrate at a rate of 2 Å/s. Five colours can be extracted out in the Baishi's peony painting displayed in this work: black, green, yellow, red and pink. To generate different colours in the painting, five masks were utilized to prepare LiF spacer layers with different thickness, as demonstrated in Fig S13. LiF films with different thickness were deposited on the surface of Ag layer by performing thermal evaporation at a deposition rate of 1 Å/s. The thickness of LiF film in the five regions was 60 nm, 360 nm, 430 nm, 500 nm and 550 nm, respectively. Then, Ag clusters were deposited on the surface the Ag film/LiF structure by performing gas-phase cluster beam deposition at a deposition rate of 0.5 Å/s and the deposition time is 10 min.

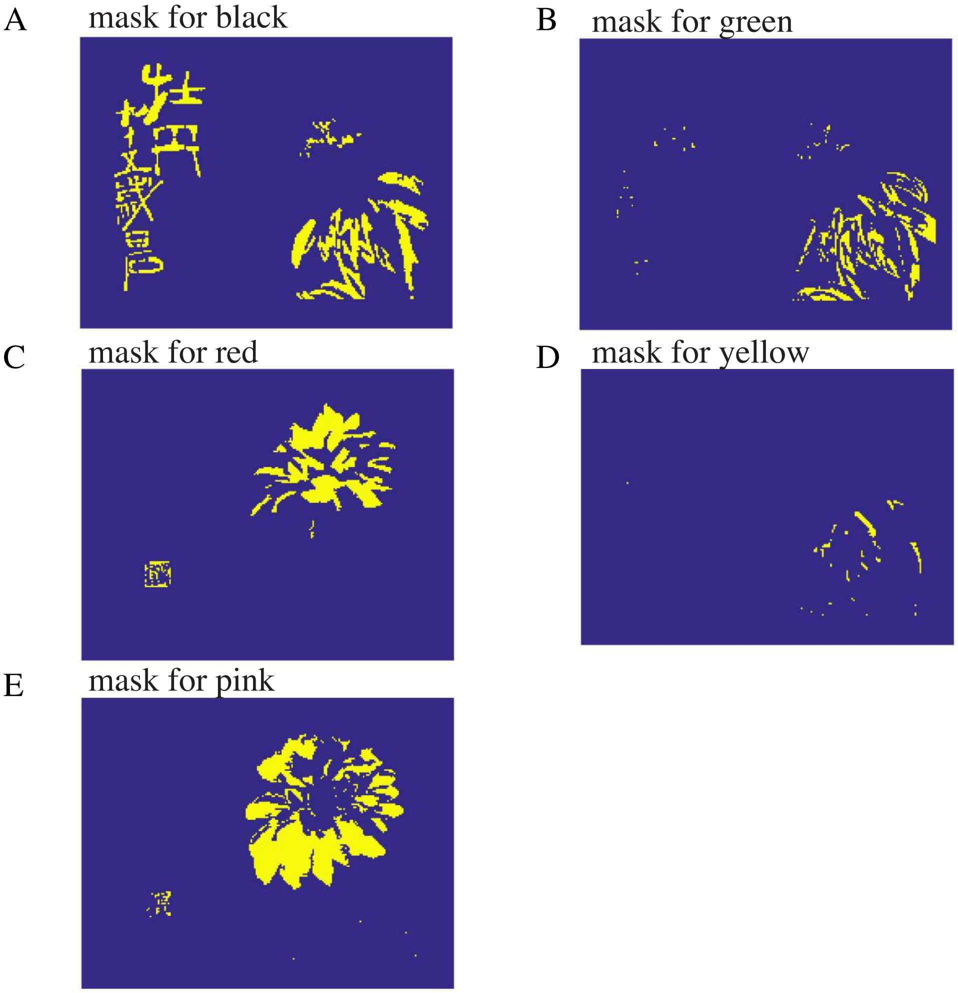

Supplementary Figure 13: Designed masks for the painting.

Supplementary Note 11: Reflection at oblique angles

We first investigate the situation with incident light at oblique angles, as demonstrated in Fig.S14A. The spectra with different incident angle θ_{inc} are summarised in Fig. S14B. Due to the sensitivity of the spacer effective thickness, the reflection deviates from normal incident as θ_{inc} increases. The spectrum maintains its feature when $\theta_{inc} < 20^o$, which is further clarified by images of the reflected light beam demonstrated in Fig. S14C.

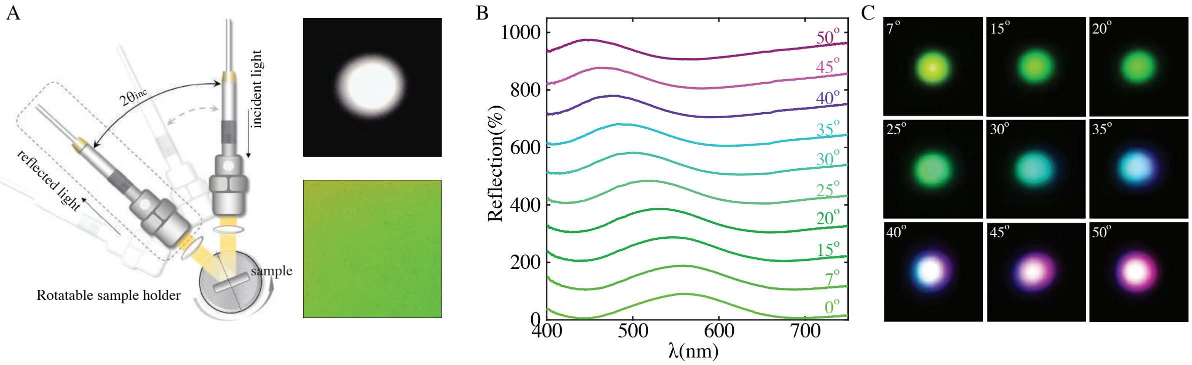

Supplementary Figure 14: (A) The setup of the measurement. A white light source (top-right panel) shines on the sample (bottom-right panel), with incident angle θ_{inc}. Specular reflection is collected. (B) The reflection spectra with different values of θ_{inc}. (C) Images of the reflected light beam with different values of θ_{inc}.

The diffusive reflection under the normal incidence of the sample is also measured, with the setup illustrated in Fig.S15A. The reflection spectra shown in Fig.S15B are normalised to exclude the attenuation. The spectra of the diffusive light at different angles remain almost the same. The spectrum beyond $\theta_c > 6^o$ is not available due to the minute intensity.

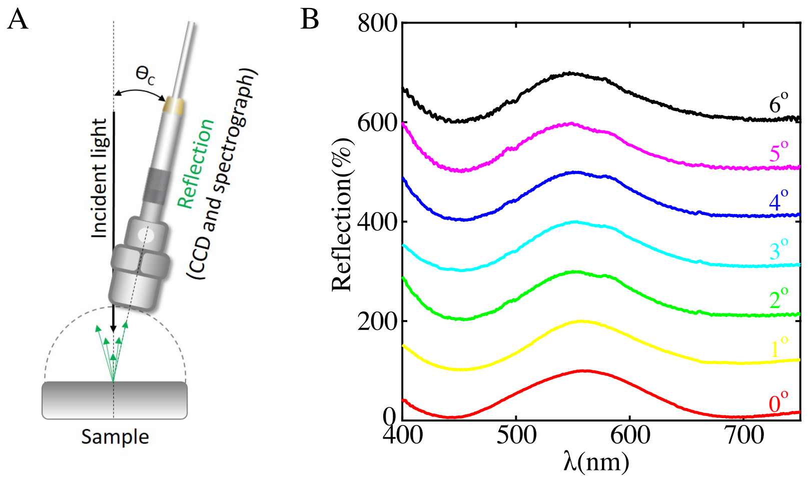

Supplementary Figure 15: (A) The setup of the measurement. A white light source shines normaly on the sample and diffusive reflection is collected at different angles θ_c. (B) The reflection spectra at different θ_c.

Supplementary Reference

1. Haus, H. A. *Waves and fields in optoelectronics* (Prentice-Hall,, 1984).

2. Kats, M. A., Blanchard, R., Genevet, P. & Capasso, F. Nanometre optical coatings based on strong interference effects in highly absorbing media. *Nature materials* **12**, 20 (2013).

3. Galinski, H. *et al.* Scalable, ultra-resistant structural colors based on network metamaterials. *Light: Science & Applications* **6**, e16233 (2017).

4. Kristensen, A. *et al.* Plasmonic colour generation. *Nature Reviews Materials* **2**, 16088 (2017).

5. Han, M. *et al.* Controllable synthesis of two-dimensional metal nanoparticle arrays with oriented size and number density gradients. *Advanced Materials* **19**, 2979–2983 (2007).

6. Narasimhan, V. *et al.* Multifunctional biophotonic nanostructures inspired by the longtail glasswing butterfly for medical devices. *Nature nanotechnology* **13**, 512 (2018).

7. Siddique, R. H., Gomard, G. & Hölscher, H. The role of random nanostructures for the omnidirectional anti-reflection properties of the glasswing butterfly. *Nature communications* **6**, 6909 (2015).

8. Wyszecki, G. & Stiles, W. S. *Color science*, vol. 8 (Wiley New York, 1982).

9. Schanda, J. *Colorimetry: understanding the CIE system* (John Wiley & Sons, 2007).

10. Liu, C. *et al.* Enhanced energy storage in chaotic optical resonators. *Nature Photonics* **7**, 473 (2013).
